# Thread Embedding Acupuncture Inhibits Ultraviolet B Irradiation-Induced Skin Photoaging in Hairless Mice

**DOI:** 10.1155/2015/539172

**Published:** 2015-06-22

**Authors:** Yoon-Jung Kim, Ha-Neui Kim, Mi-Sook Shin, Byung-Tae Choi

**Affiliations:** ^1^Department of Korean Medicine, School of Korean Medicine, Pusan National University, Yangsan 626-870, Republic of Korea; ^2^Department of Korean Medical Science, School of Korean Medicine, Pusan National University, Yangsan 626-870, Republic of Korea; ^3^National Assembly of the Republic of Korea, Oriental Medical Clinic, Seoul 156-701, Republic of Korea; ^4^Korean Medical Science Research Center for Healthy-Aging, Pusan National University, Yangsan 626-870, Republic of Korea

## Abstract

Thread embedding acupuncture (TEA) is an acupuncture treatment applied to many diseases in Korean medical clinics because of its therapeutic effects by continuous stimulation to tissues. It has recently been used to enhance facial skin appearance and antiaging, but data from evidence-based medicine are limited. To investigate whether TEA therapy can inhibit skin photoaging by ultraviolet B (UVB) irradiation, we performed analyses for histology, histopathology, in situ zymography and western blot analysis in HR-1 hairless mice. TEA treatment resulted in decreased wrinkle formation and skin thickness (Epidermis; *P* = 0.001 versus UV) in UVB irradiated mice and also inhibited degradation of collagen fibers (*P* = 0.010 versus normal) by inhibiting proteolytic activity of gelatinase matrix-metalloproteinase-9 (MMP-9). Western blot data showed that activation of c-Jun N-terminal kinase (JNK) induced by UVB (*P* = 0.002 versus normal group) was significantly inhibited by TEA treatment (*P* = 0.005 versus UV) with subsequent alleviation of MMP-9 activation (*P* = 0.048 versus UV). These results suggest that TEA treatment can have anti-photoaging effects on UVB-induced skin damage by maintenance of collagen density through regulation of expression of MMP-9 and related JNK signaling. Therefore, TEA therapy may have potential roles as an alternative treatment for protection against skin damage from aging.

## 1. Introduction

Acupuncture, one of the main medical practices in oriental medicine, is traditionally performed by placing disposable needles into specific points of the body and is also widely used in treatment of various dermatological conditions [1]. As worldwide use of complementary and alternative medicine is significant, dermatologists are seeking information on the alternative treatment strategy [2]. Thread embedding acupuncture (TEA) is another type of acupuncture in which thread is inserted at specific points for cure of various disorders. Recently, TEA therapy targeting specific points on the facial muscle has confirmed the efficacy for wrinkles and aging in modern Korean medical clinics.

Ultraviolet (UV) A (320–400 nm) and UVB (280–320 nm) can reach the earth's surface and consequently be absorbed by human skin; thus, only these two types of UV are of environmental significance [3, 4]. However, UVB is the most biologically damaging UV type in skin [5]. UVB wavelengths can penetrate the epidermis layer and are almost completely absorbed in the upper dermis, where it damages residing skin keratinocytes and dermal fibroblasts [6, 7]. UVB-induced damage is cumulative and causes pronounced changes in the appearance and structure of the skin, namely photoaging [5, 8, 9]. UV-induced photoaging of the skin is characterized by various pathological changes including thickening, wrinkling, pigmentation, and dryness [10, 11]. Wrinkle formation of skin is a notable feature of photoaging and matrix metalloproteinases (MMPs) are key enzymes in these formations [10].

MMPs expression due to UV exposure contributes to wrinkle formation through dermal destruction of the basement membrane followed by degradation of extracellular matrix components, including collagen and elastin fibers [4, 12]. Previous studies focused on the collagenase MMP-1 and gelatinase MMP-9, which are used as major markers of UV-induced photoaging [12, 13]. In particular, MMP-9 induced by UV plays an important role in the final degradation of cleaved collagen into gelatin and small peptides after initial cleavage by collagenases [4, 14, 15]. Therefore, blocking MMP-9 and related signaling molecules may be additional targets to prevent photoaging of UVB irradiation. UV irradiation generates increased reactive oxygen species (ROS) levels in the skin [16] and amplifies the signals leading to activation of mitogen-activated protein kinases (MAPKs) and phosphatidylinositol-3-kinase (PI3K)/Akt [4, 8, 17]. These kinases ultimately stimulate the transcriptional activities of activator protein-1 (AP-1) and nuclear factor (NF) *κ*B encoding for MMPs [18, 19].

Cosmetic acupuncture is performed to enhance facial skin appearance such as rejuvenation of the face and treatment of wrinkles [20]. TEA therapy is considered a useful clinical treatment as cosmetic acupuncture in Korea, but clinical data from evidence-based medicine are limited. In addition, no experimental studies describing cutaneous effects and their molecular mechanisms have been reported. A hairless mouse model is suitable for study of photoaging such as wrinkle formation by UV irradiation. Therefore, we investigated the potential effects of TEA and its molecular mechanism on UVB-induced skin damage by examining the levels of thickness and density of collagen fibers, expression of MMP-9 and its signaling in skin of hairless mice.

## 2. Materials and Methods

### 2.1. Animals

Female hairless mice (HR-1, *n* = 20; age: 8 weeks; weight: 22–25 g) were obtained from Dooyeol Biotech (Seoul, Korea) and acclimated for 1 weeks. The mice were housed at 22°C with 12 h cycles of dark and light and were fed a commercial diet and allowed tap water ad libitum throughout the study. All animal experiments were conducted with approval of Pusan National University Animal Care and Use Committee (approval number PNU 2014-0517). The mice were randomly divided into 4 groups, normal, TEA alone treated, UV alone irradiated, and UV irradiated plus TEA treated mice (UV + TEA). Each group consisted of five rats.

### 2.2. UV Irradiation

A UV lamp (Philips, Somerset, NJ, USA) with emission spectrum between 290 and 315 nm was used for UV irradiated mice. Mice were anesthetized with 8% chloral hydrate by i.p. injection and were positioned 30 cm above the mouse dorsal area. A UV meter (Lutron UV-340A, Taipei, Taiwan) was used for measurement of UV irradiance. A UV light was applied to the mice for 5 weeks, and the amount of irradiance was gradually increased from 1 MED up to 4 MED (1 MED = 1 minimal erythema dose = 100 mJ/cm^2^) with no injury. The dorsal skin of mice was exposed to UV light three times per week at 1 MED for the first week, then at 2 MED three times per week for two weeks, and at 4 MED two times per week for the last two weeks.

### 2.3. Treatment of TEA

The medical procedure for TEA, which was provided by Triple-C Medical (Bi-Poly, Lot 121102-1, Seoul, Korea), consists of two parts, needle and thread (polydioxanone suture thread). For the TEA treated mice, the needle is used for application of acupuncture and easily embedding thread in target locations. Absorbable thread is a major part of the treatment of embedding acupuncture, which is buried in the dorsal skin of mice. Gauge and length of the needle is 29 G × 12.7 mm, and thread length is 50 mm. Threads were embedded to the 4 spots of the dorsal area based on the posterior midline per one mouse at the beginning of the second week after UV irradiation under 8% chloral hydrate anesthesia. Two of them were treated at upward pelvis level (L4), the other half downward pelvis at 3 mm each apart.

### 2.4. Wrinkle Measurement

Skin condition was confirmed visually by taking pictures of the mouse dorsal skin at the end of every week. Just before mice were sacrificed, replicas of mouse dorsal skin were prepared using a Silflo kit (CuDerm Corporation, Dallas, TX, USA) to measure depth and width of wrinkles. Skin silicon impressions were analyzed using Visioline VL650 (Courage & Khazaka, Cologne, Germany).

### 2.5. Histological Analysis and Collagen Staining

Dorsal skins were obtained from sacrificed mice under anesthesia at the end of the experiment. Skins were fixed in 4% paraformaldehyde and 20 *μ*m thick cryosections were prepared. Haematoxylin and eosin staining was performed to examine epidermal and dermal thickness. Masson's trichome staining was performed using a Masson's trichrome staining kit (Abcam, Cambridge, MA, USA) to examine density of collagen fibers. After dehydration, slides were mounted in the mounting medium (Vector Laboratories, Burlingame, CA, USA) and captured using a laser scanning optical microscope Axio Vision LSM 510 (Carl Zeiss Inc., Gottingen, Germany). IMT i-solution (IMT i-solution Inc., Vancouver, BC, Canada) was used for automatic measurement for analysis of its staining properties.

### 2.6. In Situ Zymography and Immunohistochemistry

In situ zymography was used to determine the activity and location of gelatinase. Skin tissue was not fixed and rapidly frozen with 2-methylbutane and liquid nitrogen. The 20 *μ*m thick cryosections were prepared, followed by reaction using a Molecular Probes' EnzChek Gelatinase Assay Kit (Life Technologies, Eugene, OR, USA) at 37°C for approximately 8 h in dark humidified chambers. After washing, skins were fixed in 10% neutral buffered formalin in the dark. To evaluate MMP-9 colocalization, sections were incubated with primary anti-MMP-9 (Millipore, Billerica, MA, USA) in antibody dilution buffer (1x PBS, 1% BSA, 0.3% Triton X-100) and then washed with PBS overnight in the dark. The sections were incubated in corresponding secondary antibody goat anti-rabbit IgG-TR (Vector Laboratories) for 2 h at room temperature in the dark, followed by washing with PBS. For DAPI staining, sections were incubated with DAPI (Vector Laboratories) in PBS for 10 min, and then slides were mounted in fluorescence-mounting media (Vector Laboratories). Optical microscope Axio Vision LSM 510 (Carl Zeiss Inc.) was used for measuring the fluorescence intensity.

### 2.7. Western Blot Analysis

Tissues were washed in cold HEPES buffer and homogenized in lysis buffer. Equal amounts of total proteins were separated by 10–12% sodium dodecyl sulfate polyacrylamide gel electrophoresis, and transferred to a nitrocellulose membrane (Whatman, Dassel, Germany). Immediately, membranes were blocked with 5% nonfat milk in PBST containing 0.4% Tween-20. Membranes were incubated with primary antibodies recognizing ERK (Cell Signaling Technology, Danvers, MA, USA), phospho-ERK (pERK, Cell Signaling Technology), JNK (Cell Signaling Technology), phospho-JNK (pJNK, Cell Signaling Technology), Akt (Cell Signaling Technology), phospho-Akt (pAkt, Cell Signaling Technology), NF-*κ*B (Santa Cruz Biotechnology Inc., CA, USA), and MMP-9 (Millipore) for 1-2 h at room temperature, and then incubated with secondary antibody. *β*-actin (Sigma-Aldrich, St. Louis, MO, USA) was used as a loading control for all experiments. Secondary antibodies were purchased from Santa Cruz Biotechnology (Santa Cruz, CA, USA). Quantification of immunoreactivity corresponding to the total bands was confirmed by densitometric analysis using an Image Quant LAS 4000 (Fujifilm, Tokyo, Japan).

### 2.8. Statistical Analysis

Results are expressed as the mean ± SD. The Sigmastat statistical program Version 11.2 (Systat Software, SanJose, CA, USA) was used for statistical analysis of data. The *t*-test was used for comparison of differences between groups. A value of *P* < 0.05 was considered statistically significant, and exact *P* values were shown unless *P* < 0.001.

## 3. Results

### 3.1. Effects of TEA on Wrinkle Formation and Skin Thickness in UVB Irradiated Hairless Mice

**Figure 1:**
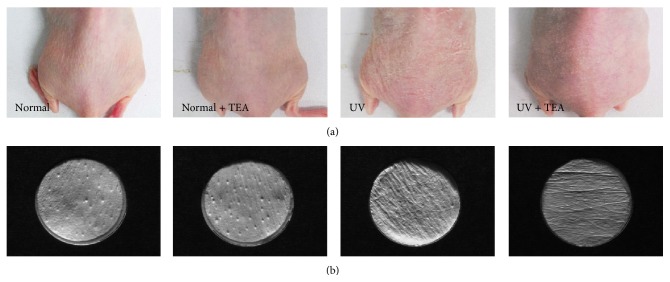
Visible skin condition and wrinkle formation in dorsal skin of TEA-treated mice against UVB-induced skin damage. (a) Photographs and (b) replicas of mouse dorsal skin were taken to show skin conditions. TEA treatment resulted in better visible skin condition and alleviated wrinkle formation in UV irradiated mice. TEA, thread embedding acupuncture; UV, ultraviolet B.

**Figure 2:**
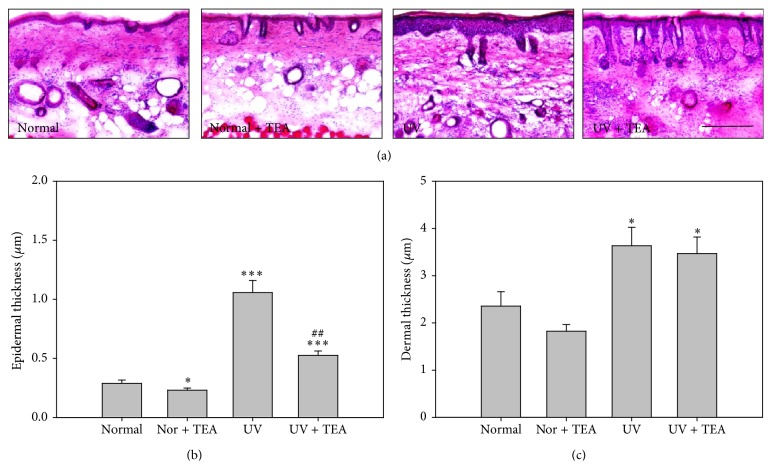
Histological analysis for epidermal and dermal thickness in dorsal skin of TEA-treated mice against UVB-induced skin damage. (a) Haematoxylin and eosin staining and its histogram estimated for (b) epidermal and (c) dermal thickness. TEA treatment significantly suppressed increase in epidermal thickness and decrease in dermal thickness in UV irradiated mice. ^*∗*^ *P* < 0.05 and ^*∗∗∗*^ *P* < 0.001 versus normal mice; ^##^ *P* < 0.05 versus UV alone irradiated mice. Scale bars = 200 *μ*m. Nor, normal; TEA, thread embedding acupuncture; UV, ultraviolet B.

Visible skin conditions were identified by photographs taken at the end of every week during the experiment (Figure 1(a)). Compared with normal mice, the skin of UV irradiated mice was dry, rough, and flaky. However, less flakiness and roughness were observed for UV + TEA mice than UV alone irradiated mice. After application of TEA for 4 weeks, wrinkle formation was further analyzed by dorsal skin silicon replica (Figure 1(b)). The wrinkles of normal mice were thin and shallow, while those of UV-irradiated mice showed thick and deep wrinkles. UV + TEA mice showed wrinkle recovery in percentage of wrinkle area, wrinkle length, and wrinkle depth as compared with UVB alone treated mice, however no significant changes were observed. Next, we investigated the photoprotective effects of TEA by examining histological changes in the skin (Figures 2(a) and 2(b)). Thicker epidermis (*P* < 0.001 versus normal group) and dermis (*P* = 0.011 versus normal group) were observed in UVB-irradiated mice compared to those of normal. However, UV + TEA mice showed marked recovery from these pathological changes (Epidermis; *P* = 0.001 versus UV group) as compared with UVB alone treated mice. Results showing attenuation of UVB-induced wrinkle formation and skin thickening suggest that TEA may have protective effects against UVB-induced skin damage.

### 3.2. Effects of TEA on Collagen Fiber Loss and Proteolytic Activity of MMP-9 in UVB Irradiated Hairless Mice

**Figure 3:**
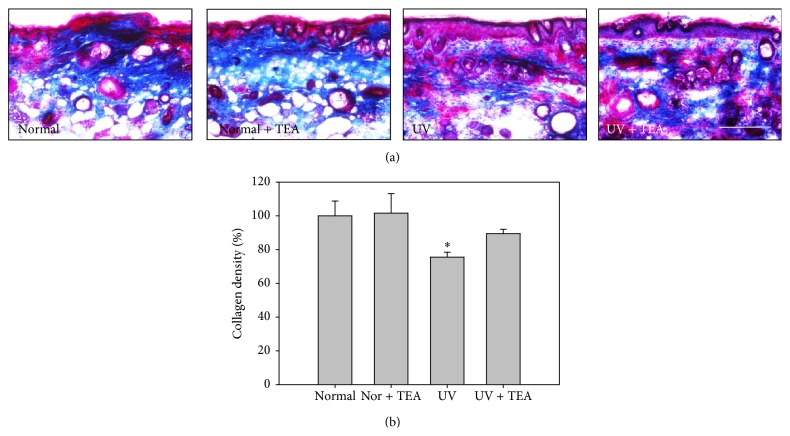
Analysis for density of collagen fibers in dorsal skin of TEA-treated mice against UVB-induced skin damage. (a) Masson's trichrome staining and (b) its histogram estimated for relative collagen density. TEA treatment significantly prevented UVB induced loss of collagen fibers. ^*∗*^ *P* < 0.05 versus normal mice. Scale bars = 200 *μ*m. Nor, normal; TEA, thread embedding acupuncture; UV, ultraviolet B.

**Figure 4:**
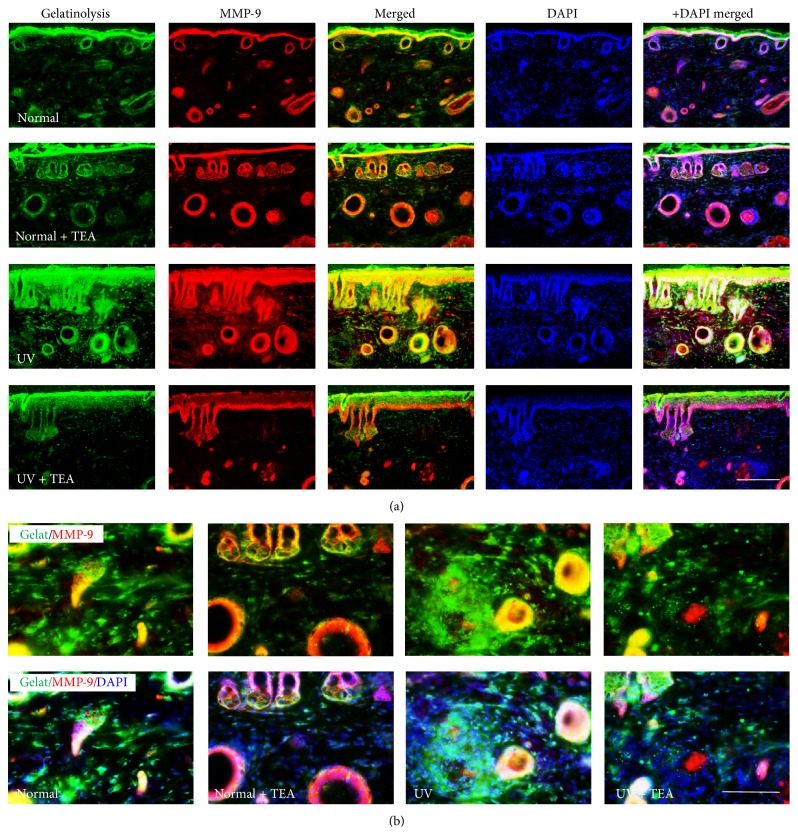
In situ zymographic analysis for MMP-9 activity in dorsal skin of TEA-treated mice against UVB-induced skin damage. (a) Images of gelatinoylysis/MMP-9 and (b) its higher magnification. Degradation of collagen by gelatinases was detected as fluorescence. TEA treatment significantly suppressed gelatinoylysis and MMP-9 expression in the dermal region. TEA, thread embedding acupuncture; MMP-9, matrix-metalloproteinase-9; UV, ultraviolet B. Scale bars = 200 *μ*m (a) and 100 *μ*m (b).

Masson's trichrome staining and in situ zymography were performed in order to observe changes in density of collagen fiber and enzyme activity of MMP-9. Masson's trichrome staining (Figures 3(a) and 3(b)) showed a significant decrease in abundance and density of collagen fiber (*P* = 0.010 versus normal) in the dermis region of the skin in UVB irradiated mice compared to normal mice. However, treatment with TEA inhibited loss of collagen fiber induced by UVB irradiation. Because gelatinase MMP-9 plays an important role in the final degradation of collagen fibers, the activity and location of MMP-9 were analyzed by in situ zymography (Figure 4). In situ zymography analysis generally showed gelatinolytic activity colocalized with the MMP-9 reaction, and marked fluorescence was mainly localized in keratinocytes of epidermis and hair follicles in normal mice. The fluorescence properties of UVB irradiated mice are greater than those of UVB nonirradiated mice, particularly for gelatinolysis in cell components of dermis. However, UVB irradiated mice treated with TEA showed a significant decrease of fluorescence intensity of gelatinolysis compared with UVB alone irradiated mice, which showed an inhibited proteolytic activity of MMP-9 by TEA. These results suggest that TEA exerts preventive effects in degradation of collagen fiber through inhibition of proteolytic activity of gelatinase MMP-9 against UVB-induced skin damage.

### 3.3. Effects of TEA on Expression of MMP-9-Related Signaling in UVB Irradiated Hairless Mice

**Figure 5:**
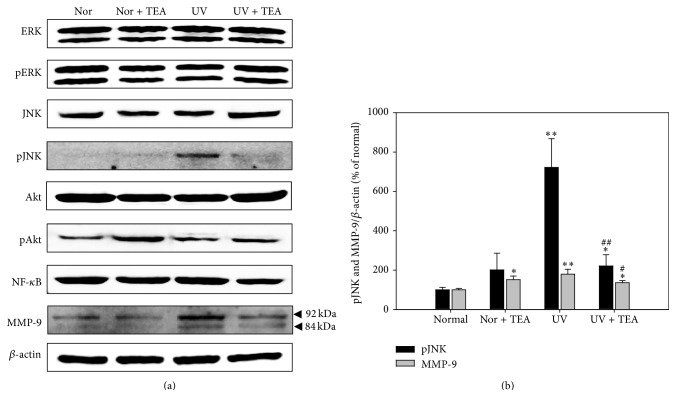
Western blot analysis for MMP-9 and its related signaling factors in dorsal skin of TEA-treated mice against UVB-induced skin damage. (a) Western blotting and (b) its relative densities of proteins. Relative densities of proteins of *β*-actin were quantified. UVB irradiation significantly increased expression of pJNK and MMP-9, but TEA treatment resulted in recovery to that of normal mice. ^*∗*^ *P* < 0.05 and ^*∗∗*^ *P* < 0.01 versus normal mice; ^#^ *P* < 0.05 and ^##^ *P* < 0.01 versus UV-B irradiated mice. Abbreviations: Nor, normal; TEA, thread embedding acupuncture; ERK, extracellular regulated signal kinase; JNK, c-Jun N-terminal kinases; NF-*κ*B, nuclear factor-kappa B; MMP-9, matrix-metalloproteinase-9; UV, ultraviolet B.

We performed Western blot analysis to examine the effects of TEA on expression of UVB-induced MMP-9 and its related signaling pathway factors (Figure 5). For key activator of UVB-induced cellular responses including extracellular signal-regulated kinase (ERK), Jun N-terminal kinase (JNK) and Akt, there were no significant changes in expression of total proteins. However, mice exposed to UVB exhibited a marked increase in expression of pJNK (*P* = 0.002 versus normal group) which was attenuated by TEA treatment (*P* = 0.005 versus UV group). A slight increase of pAkt was also observed in TEA alone treated mice and NF-*κ*B in UV alone irradiated mice; however, no significant changes were observed. Expression of MMP-9 was significantly increased by UVB irradiation (*P* = 0.006 vs. normal group), but also decreased by TEA treatment (*P* = 0.048 vs. UV group). As a whole, the expressions of pJNK and MMP-9 by UV irradiation were recovered by TEA treatment like normal mice. These results indicate that TEA treatment exerts protective activity against UVB-induced skin damage by regulating MMP-9 expression through inhibition of JNK activation.

## 4. Discussion

Recently, many new products and medical care have arisen that are useful in the rejuvenation of facial skin [21]. Like traditional acupuncture for dermatological conditions [22], TEA is also used for facial skin appearance such as rejuvenation of the face and treatment of wrinkles, and we thus hypothesize the antiphotoaging activity of TEA seen in UV irradiated mice due to inhibition of skin damage. We evaluated, for the first time, the protective efficacy and its molecular factors of TEA against skin photoaging in UVB irradiated hairless mice. Our work provides clear evidence that TEA treatment is effective in suppressing signs of photoaging including epidermal thickness and collagen fiber loss. In addition, TEA exerts its photoprotective effects against UVB irradiated skin damage by inhibiting JNK activation with subsequent reduction in MMP-9 expression. We therefore conclude that TEA treatment may be useful therapy for prevention of UVB-induced skin photoageing.

UVB-induced photoageing is a cumulative process, but shares the same mechanisms in part as intrinsic ageing [14]. UV exposure affects internal changes of the skin structure. UVB irradiated skin showed thicker epidermis by increasing thickness of stratum corneum [23]. Collagen fibers are a major component of the connective tissue in skin involved in the maintenance of dermal strength and elasticity and its loss of amount is an important step leading to skin aging [10, 24]. In the current study, visible conditions and wrinkle formation by skin replica analysis were improved by TEA treatment in UVB irradiated mice. However, compared to other studies, we used weaker and shorter exposure to UVB; thus, skin replica analysis showed no significant changes. Next, we performed histological and histopathological analysis. TEA treated mice showed a significantly thinner epidermal layer and compacted collagen fibers compared to those of UVB-irradiated mice. These results suggest that TEA may protect against UVB-induced skin damage by balancing epidermal thickness and maintaining collagen density.

As major matrix-degrading enzymes, MMPs share a wide range of substrates and can degrade various components of extracellular matrix, including collagen fiber [14, 25]. Several types of MMPs are required in stage of collagen loss in UV irradiation. Collagenases MMP-1 and MMP-3 play a prominent role in initiating degradation of type I, III, and IV collagens, major components of the skin dermis [13]. Gelatinase MMP-9 plays a major role in the final degradation of cleaved collagen into gelatin and small peptides, a major factor responsible for wrinkle formation [4, 8, 14]. UVB exposure induces overexpression of MMP-9 and both wrinkle formation and skin thickness are associated with this enzyme [12, 26]. Excessive MMPs in the skin are secreted from UV-stimulated keratinocytes, dermal fibroblasts, and inflammatory cells contributing to degradation of extracellular matrix in photoaged skin [27–29]. UVB irradiation induced significant gelatinolytic activity and MMP-9 reaction, but the gelatinolytic activity and MMP-9 reaction in the dermis were significantly reduced by treatment with TEA, suggesting that TEA inhibits gelatinolytic activity of MMP-9. Our results suggest that TEA ameliorates the gelatinolytic activity of MMP-9 and subsequent alleviation of collagen loss.

Thus, we attempted to detect the MMP-9-related cellular signaling pathway on UVB irradiated mice. UVB irradiation generates reactive oxygen species (ROS) in the skin, which stimulates the major signaling leading to photoaging [16]. ROS induced by chronic UVB exposure activates signaling pathways to activate kinases such as MAPKs and PI3K/Akt [8, 17, 30]. These activated kinases ultimately stimulate the transcriptional activities of AP-1 and nuclear factor (NF) *κ*B, the genes encoding for MMPs [18, 19]. In our western blot analysis, level of pJNK and MMP-9 by UVB was significantly reduced by TEA treatment. NF-*κ*B is also slightly downregulated in TEA treated UV irradiated mice and pAkt was increased in normal mice; however, there were no significant differences. Therefore, UVB-induced phosphorylation of JNK may be an important molecular factor for controlling MMP-9 expression by TEA treatments. TEA treatment resulted in reduced activation of JNK and induction of transcription factor NF-*κ*B in UVB irradiated mice and subsequent decreases in the production of MMP-9 in skin.

In Korea, TEA therapy has been applied clinically for many disorders, even in the fields of cosmetic surgery and aging. Korean medical practitioners assume that embedded thread at the targeted point works as a prolonged stimulator of tissues, while traditional acupuncture involves a relatively short timeframe for receiving treatment. Raw materials of thread have been used in absorbable animal tissues, drugs, or suture threads. Thus, there are some merits to application of this therapy to various dermatological conditions, because it can be naturally absorbed safely into the body with passage of time. Despite its medical usage, exact mutual relation between this thread and surrounding tissues was not fully determined because of lack of previous studies. However, facial augmentation using various implants such as silhouette sutures and cog thread can achieve successful facial appearance and reinforce the soft tissue of the face [31–33].

As the suture thread is dissolved by slow hydrolysis in the presence of tissue fluid, exogenous materials are surrounded by a capsule of fibrosis over a period of months [34, 35]. Fibrosis is predominantly characterized by excessive deposition of extracellular matrix, especially collagen [36]. JNK is generally considered to be antifibrotic and MMPs participate in extracellular matrix degradation [36, 37]. Further studies of TEA therapy on the wrinkle formation under photoaging are necessary in order to establish a more accurate mechanism. However, TEA may have played a role in tightening lax skin and reform the soft tissue of the face as long lasting stimulator for collagen fiber formation in the fibrosis, due to the downregulation of JNK and MMP-9 activity.

Consequently, we observed first that antiphotoaging effects of TEA treatment are exerted by maintenance of collagen density, reduction in expression of pJNK and of MMP-9, which was induced by regulation of activated JNK, and subsequent expression of MMP-9. Our results provide some basis for beneficial effect of TEA therapy against UV induced photoaging and it may be useful as an alternative treatment for protection against skin damage such as wrinkle and photoaging.

## Abbreviations

TEA: Thread embedding acupuncture
UVB: Ultraviolet B
ERK: Extracellular signal-regulated kinase
JNK: c-Jun N-terminal kinase
MMP: Matrix metalloproteinases
ROS: Reactive oxygen species
MAPK: Mitogen-activated protein kinases
AP-1: Activator protein-1
NF: Nuclear factor.
